# Sharing your work by self-archiving: encouragement from the *Journal of the Medical Library Association*

**DOI:** 10.5195/jmla.2020.877

**Published:** 2020-01-01

**Authors:** Abigail Goben, Katherine G. Akers

**Affiliations:** Library of the Health Sciences, University of Illinois at Chicago, Chicago, IL, agoben@uic.edu; Editor-in-Chief, *Journal of the Medical Library Association*, JMLA@journals.pitt.edu

## Abstract

Self-archiving offers opportunities for authors to more broadly disseminate their work—both in pre-print form before its submission to a journal and in post-print form after its acceptance and publication in a journal. This editorial provides authors with guidance in navigating the rapidly changing options for self-archiving and affirms that the *Journal of the Medical Library Association* encourages authors to self-archive their work to boost its reach and impact.

As scholarly publishing continues to evolve, authors now have opportunities to openly share their manuscripts online to receive informal feedback from colleagues before submitting them to peer-reviewed journals and to enhance their dissemination after journal acceptance. Although the *Journal of the Medical Library Association (JMLA)* has embraced its open access status for nearly twenty years [1, 2], the *JMLA* team also recognizes that authors have other avenues through which to share their scholarly work and we encourage authors to ask the community for feedback on their manuscripts prior to their submission.

Self-archiving by sharing online pre- or post-print versions of manuscripts offers opportunities to engage in early conversations about manuscripts, to solicit critical feedback before their submission to journals, and to improve their accessibility to readers [3, 4]. In addition to facilitating scholarly discourse and potentially improving the strength of their manuscripts, self-archiving one’s work allows librarians and information professionals to gain firsthand knowledge of the intricacies of scholarly communication through direct participation in emerging practices.

Engaging in self-archiving can introduce authors to unfamiliar jargon and a multitude of options that must be carefully considered in order to make informed decisions regarding which version of their manuscripts to share, when and where to share their manuscripts, and how to link their manuscripts to the publisher’s versions of record [5]. This editorial provides a brief guide to self-archiving for health sciences librarians and information professionals and emphasizes *JMLA*’s supportive stance on self-archiving.

## TIPS FOR SELF-ARCHIVING

### Selecting which version to share

**Figure 1:**
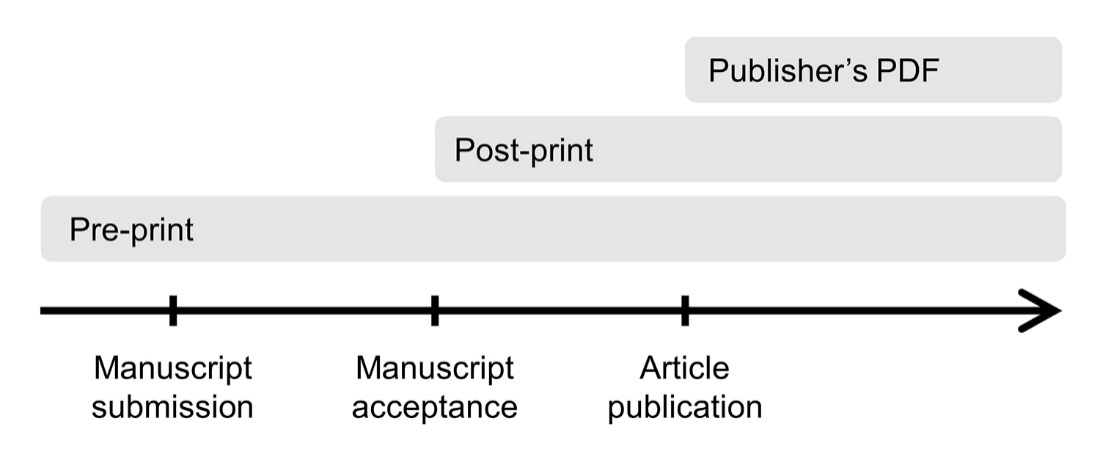
Versions of a manuscript that can be self-archived at different stages of the writing and publishing process

When self-archiving, it is important to choose which version of the manuscript to share and when to share it during the writing and publishing process (Figure 1). Most journals have policies regarding whether, when, and where authors can post various versions, though these can also be negotiated by authors using the Scholarly Publishing and Academic Resources Coalition (SPARC) author addendum [6] or alternates.

- **Pre-print:** A pre-print is a version of a manuscript before it is submitted to a journal [7]. Sharing a pre-print can be a good way to solicit feedback from readers before formal peer review. However, some journals consider a pre-print a form of prior publication and may, thus, be unwilling to consider the manuscript for publication. It is important to check the policies of target journals before publicly sharing a pre-print, and it is good practice to state in the journal submission cover letter whether a pre-print of the manuscript has been made publicly available.
- **Post-print:** A post-print is the final, accepted version of a manuscript before it is typeset by the journal for publication [7]. It incorporates all revisions made during the peer and/or editorial review process. In addition to authors sharing a post-print, journals may release the post-print version of a manuscript before its formal publication; in PubMed, these appear with the designation “Epub ahead of print.”
- **Publisher’s PDF:** A publisher’s portable document format (PDF) is a professionally copyedited, professionally typeset, and formally published version of an article. This seems to be the least common version for self-archiving, although the proportion of journals that explicitly allow self-archiving of the publisher PDF is unknown. An example of this policy is from the *Journal of Dental Education*, which requires archiving only of the publisher’s PDF without embargo [8].

### Identifying journal self-archiving policies

When self-archiving, it is essential to identify and understand the policies of journals to which one plans to or has already submitted a manuscript. To help accomplish this, the SHERPA/RoMEO resource aggregates the self-archiving and copyright policies of more than 22,000 journals. Searching SHERPA/RoMEO for a specific journal title yields information such as whether the journal allows self-archiving of pre-prints, post-prints, and publisher’s PDFs; where these manuscript versions can be posted; whether their release should be embargoed; and how they should be linked to the final publisher’s version of record. To confirm the information found in SHERPA/RoMEO, authors should also consult the websites of their target journals.

### Selecting a location for self-archiving

Authors have an increasing wealth of locations to self-archive their manuscripts.

- **Disciplinary repositories:** Two repositories in the field of library and information science (LIS) currently exist: LIS Scholarship Archive (LISSA) and e-LIS. LISSA is hosted on the Open Science Framework (OSF) and allows a variety of deposit types, including manuscripts, posters, data sets, and code. e-LIS is hosted by the Library System of the University of Naples Federico II and primarily serves as a repository of manuscript pre-prints, post-prints, and technical papers. Neither repository charges a deposit fee. Although PubMed Central (PMC) currently serves as the largest disciplinary repository for biomedical journal articles, including those in health sciences librarianship and information science journals, it strictly houses accepted versions of manuscripts (post-prints or publishers’ PDFs) that are published in PMC-participating journals (including *JMLA*) or supported by National Institutes of Health funding.
- **Institutional repositories:** Many academic libraries have an institutional repository (IR) that aggregates and preserves the scholarly output of their institutions and facilitates author compliance with open or public access requirements. IRs predominantly house manuscripts, although they are increasingly being refactored or expanded to accept data, code, and other research materials. There is usually no charge for deposit in IRs, and the institution supports long-term preservation of the deposited materials.
- **General repositories:** General repositories, such as figshare and OSF, store and provide access to manuscripts and other research materials regardless of discipline or author institutional affiliation. General repositories vary in whether they are free or charge a deposit fee, and not all guarantee preservation of their contents.
- **Academic social networking sites:** Various for-profit websites, including ResearchGate and Academia.edu, aim to serve as general social networks in the academic arena. These websites encourage authors to post copies of their manuscripts for easy access and reuse by peers. Authors should understand the copyright agreements that they sign with their articles’ publishers and the restrictions therein, as many journals explicitly do not allow posting of any manuscript version on for-profit websites. Additional concerns can arise regarding lack of preservation.
- **Personal websites:** Authors can post manuscripts or publishers’ PDFs on their personal, research team, or departmental websites. This is a free option that allows authors to aggregate their works in their own web space, but it puts the obligation of hosting and managing the website directly on the author and can come at a cost to manuscript discoverability.

### Including the correct citation

If authors opt to publicly share a pre-print or post-print before or after formal publication of their articles, it is best practice to provide a citation and/or link to the publisher’s version of record with the self-archived manuscript. Some journals provide specific instructions for what type of information to include about the manuscript. In general, before the article is published, the citation should be approximated to resemble the anticipated final citation of the published article, replacing the year of publication and volume, issue, and page numbers with “in press,” “publication pending,” or “article accepted [date].” After the article is published, the self-archived manuscript should be updated with a final complete citation paired with a statement pointing readers to the publisher’s version as the official version of record.

This process can be complicated if the repository housing the self-archived manuscript assigns digital object identifiers (DOIs), as ideally each scholarly object should have only one DOI. Authors may be able to request the temporary suppression of this field in a repository and update the record with the publisher-assigned DOI upon publication. If not, the pre-print or post-print will have one DOI, and the publisher’s final version of record will have another DOI, and authors need to track both DOIs to assess their work’s impact.

### Setting an embargo

Some journals may request that authors wait until the article has been formally published before making their post-print publicly available. Accordingly, many repositories permit authors to set an embargo period during deposit that will automatically release the manuscript file on the appropriate date. This embargo allows the publisher’s version of record to be the first discoverable version and may help prevent DOI duplication issues. Other journals, such as subscription-based journals, require that authors do not publicly share their post-print until one to three years after article publication. These journals argue this delay preserves the value of the journal, but the delay could ultimately decrease article readership and impact. Making a pre-print available before manuscript submission, if allowed by the journal, could serve to preempt this requirement.

## THE JOURNAL OF THE MEDICAL LIBRARY ASSOCIATION’S SUPPORTIVE STANCE ON SELF-ARCHIVING

*JMLA* fully embraces the freedom of authors to share their work as widely as they desire to meet their own objectives—whether to obtain pre-submission feedback, increase their readership, enhance the potential impact of their work, achieve professional recognition, or support the principles of research transparency and open science.

If you publish in *JMLA*, you retain copyright and grant our publisher the right to publish your work under a Creative Commons Attribution (CC-BY) 4.0 license. You are permitted and encouraged to post a pre-print of your manuscript anywhere you choose. If your manuscript is accepted by and published in *JMLA*, we ask that you update your posted pre-print or post-print with the publisher’s assigned DOI, which can be requested from the *JMLA* editor after acceptance, and a link to the online abstract for the final published article on the *JMLA* website. No embargoes are requested or required. You are also allowed to post the publisher’s PDF anywhere you choose after publication of your article. These policies are described in greater detail on the Author Guidelines and Editorial Policies pages of the *JMLA* website and are shown in SHERPA/RoMEO’s entry for JMLA.

Health sciences librarians and information professionals who engage in scholarly publishing have the opportunity to explore, understand, and influence the availability of their own work and that of the health care professionals, researchers, and students they support. By highlighting the value of self-archiving, we emphasize *JMLA*’s dedication to open access with the ultimate goal of amplifying our authors’ voices and increasing the likelihood that their work will influence the profession’s knowledgebase and practice.

## 

**Abigail Goben**, agoben@uic.edu, http://orcid.org/0000-0002-6520-3648, Library of the Health Sciences, University of Illinois at Chicago, Chicago, IL

**Katherine G. Akers**, JMLA@journals.pitt.edu,https://orcid.org/0000-0002-4578-6575, Editor-in-Chief, *Journal of the Medical Library Association*
